# Randomized controlled trial of early arachidonic acid and docosahexaenoic acid enteral supplementation in very preterm infants

**DOI:** 10.3389/fped.2022.947221

**Published:** 2022-08-25

**Authors:** Patricia Álvarez, David Ramiro-Cortijo, María Teresa Montes, Bárbara Moreno, María V. Calvo, Ge Liu, Ana Esteban Romero, Marta Ybarra, Malaika Cordeiro, Marina Clambor Murube, Eva Valverde, Aurora Sánchez-Pacheco, Javier Fontecha, Robert Gibson, Miguel Saenz de Pipaon

**Affiliations:** ^1^Department of Neonatology, La Paz University Hospital, Universidad Autónoma de Madrid, Madrid, Spain; ^2^Department of Physiology, Faculty of Medicine, Universidad Autónoma de Madrid, Madrid, Spain; ^3^Food Lipid Biomarkers and Health Group, Instituto de Investigación en Ciencias de la Alimentación (CIAL, CSIC-UAM), Madrid, Spain; ^4^South Australian Health and Medical Research Institute, SAHMRI Women and Kids, Adelaide, SA, Australia; ^5^Department of Biochemistry, Instituto de Investigaciones Biomédicas Alberto Sols (CSIC-UAM), Madrid, Spain; ^6^SAHMRI Women and Kids, South Australian Health and Medical Research Institute, Adelaide, SA, Australia; ^7^School of Agriculture, Food and Wine, The University of Adelaide, Adelaide, SA, Australia

**Keywords:** long-chain polyunsaturated fatty acids, oxylipins, emulsion, supplementation, ARA, DHA, single-nucleotide polymorphisms

## Abstract

**Objective:**

To evaluate changes in blood long-chain polyunsaturated fatty acid (LCPUFA) and oxylipin concentrations in very preterm infants from birth to 36 weeks’ postmenstrual age (WPA) after providing an emulsified arachidonic acid (ARA):docosahexaenoic acid (DHA) supplement at two different concentrations.

**Study design:**

This prospective, randomized trial assigned infants to receive a supplement (1) 80:40 group (80 mg/kg/day ARA and 40 mg/kg/day DHA, *n* = 9) or (2) 120:60 group (120 mg/kg/day ARA and 60 mg/kg/day DHA, *n* = 9). Infants received supplement daily from birth until 36 WPA. At baseline, 21 days of life and 36 WPA, the LCPUFAs were measured in plasma by gas chromatography/mass spectrophotometry. Additionally, LCPUFAs and oxylipins were analyzed in whole blood by ultra-high-performance liquid chromatography-tandem mass spectrometry. Furthermore, a sample of oral mucosa was obtained to analyze single-nucleotide polymorphism located in the *FADS1* gene by PCR.

**Results:**

Gestational age was similar between groups (80:40 = 28^+6^ [27^+3^; 30^+3^] completed weeks^+*days*^; 120:60 = 29^+6^ [27^+3^; 30^+5^] completed weeks^+*days*^, *p* = 0.83). At 36 WPA, the change in plasma ARA was significantly different between groups (80:40 group = 0.15 [−0.67; 0.69] %nmol, 120:60 = 1.68 [1.38; 3.16] %nmol, *p* = 0.031). In whole blood, the levels of ARA-derived oxylipins (5-, 8-, 9-, 11-, 15-HETE and 8,9-EET) and EPA-derived oxylipins (18-HEPE) significantly increase from baseline to 36 WPA in the 120:60 group than the 80:40 group.

**Conclusion:**

Supplementation at high doses (120:60 mg/kg/day) increased levels of ARA, and EPA- and ARA-derived oxylipins compared to low doses (80:40 mg/kg/day). Differences were detected in EPA metabolites without a significant increase in plasma DHA.

## Introduction

Improvements in clinical practice in the neonatal intensive care unit (NICU) have increased survival rates in very preterm infants (1). Despite reduction in mortality associated with prematurity, long-term morbidities remain a concern (2). Preventive measures applied in the critical neonatal period can have a major impact, changing lifelong morbidities in these vulnerable infants.

Long-chain polyunsaturated fatty acids (LCPUFAs), particularly omega (n)-6 arachidonic acid (ARA) and n-3 docosahexaenoic acid (DHA), are crucial for infant health and neurodevelopment (3), being important modulators of inflammation. It has been reported that low blood LCPUFA levels in infants are associated with risk of neonatal morbidities, such as bronchopulmonary dysplasia (BPD) (3, 4), retinopathy of prematurity (ROP) (5), and necrotizing enterocolitis (NEC) (6, 7). In addition, the n-3:n-6 ratio has been shown as a suitable index for the neurodevelopment and risk of BPD (8, 9).

Oxylipins are PUFAs oxidation metabolites formed *via* cyclooxygenase, lipoxygenase, and cytochrome P450 pathways, synthesizing prostaglandins, thromboxanes, hydroxy- and epoxy-fatty acids, lipoxins, resolvins, protectins, and maresins (10, 11). The oxylipins are the main mediators of physiological effects of PUFAs (12). Recent developments in lipidomic methodologies have raised awareness of and interest in the large range of oxylipins. In addition, the tissue oxylipin profile does not necessarily reflect tissue fatty acid levels (13).

Very preterm infants are vulnerable to deficiency of LCPUFAs and, potentially, oxylipin imbalance. First, the shortened gestation, ∼80% of LCPUFAs selectively transferred during the last trimester of pregnancy (14). Secondly, endogenous synthesis of LCPUFAs is decreased due to immature synthesis of ARA and DHA from precursor linoleic (LA) and alpha-linoleic acids (ALA), respectively (15). Thirdly, inadequate fatty acids intake from enteral feeds (16). Furthermore, DHA levels in mother’s own milk and donors are variable (17, 18).

Currently, preterm infants receive insufficient dietary LCPUFAs (16, 17), which could impact neonatal morbidities. Clinical trials have investigated maternal and preterm infant LCPUFAs supplementation but failed to define the required dose for ARA and DHA supplementation. A previous trial using experimental lipid supplement, oil derived from fungi and microalgae, without emulsification, containing ARA:DHA (80 mg/kg/day of ARA and 40 mg/kg/day of DHA; 2:1), was not able to show different levels of ARA and DHA at 36 weeks’ postmenstrual age (WPA) (19). However, another trial with a similar ARA:DHA ratio but a higher dose (240 mg/kg/day of ARA and 120 mg/kg/day of DHA) showed differences in blood LCPUFAs concentrations, without clinical adverse outcome (20). Not only intake but also the single-nucleotide polymorphisms (SNPs) of the fatty acids desaturase 1 (*FADS1*) gene are important determinants of LCPUFAs plasma concentrations (21), considering that *FADS1* gene encodes Δ5-desaturase. This enzyme catalyzes the final step in the synthesis of ARA and eicosapentaenoic acid (EPA) (22).

We hypothesize that the benefits of ARA:DHA supplementation of premature neonates during the early perinatal stage are modulated by oxylipins. The main objective was to assess in very preterm infants the blood changes of LCPUFAs and oxylipin levels from birth to 36 WPA after providing an emulsified ARA:DHA supplement. Then, we aimed to evaluate the effects of the intervention on neonatal morbidities and brain size and function at 36 WPA.

## Materials and methods

### Study design

This prospective and randomized pilot study comparing the effect of supplementation with ARA:DHA was conducted in the NICU of La Paz University Hospital (HULP, Madrid, Spain) between October 2020 and January 2021. The trial was performed in accordance with the Declaration of Helsinki, and the protocol was approved by the HULP Ethics Committee (ref. HULP-4840).

**FIGURE 1:**
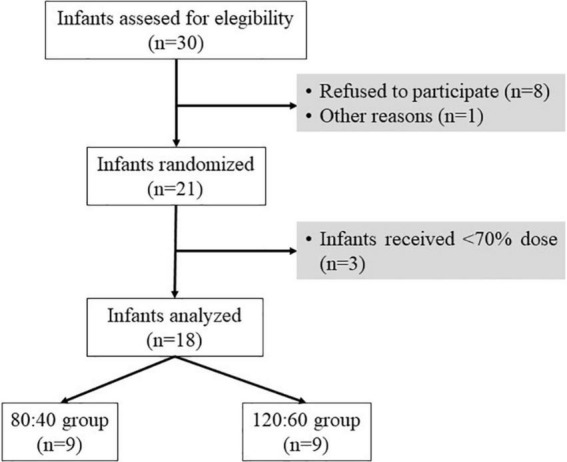
Flowchart of the allocation of eligible study infants. Sample size (*n*). Other reasons including congenital malformation or inborn errors of metabolism.

The sample size was calculated with G*Power (version 3.1.9.6, University of Kiel, Germany), considering an 80% of statistical power and a 5% of error probability. Additionally, it was considered relevant to detect, at the end of the protocol, an increase in DHA plasma levels of 3% of total fatty acids between both study groups, following the approach reported by Collins et al. (23). Consequently, the sample size per randomized group was eight very premature infants. A total of 30 infants were invited to participate in the study. The inclusion criteria were gestational age <32 weeks, admission in the NICU of HULP in the first 72 h of life, and signed informed consent by the parents or the legal representative of the newborn. The exclusion criteria were life-threatening major congenital malformation or inborn errors of metabolism, and genetic alterations. Finally, 21 preterm infants were randomized according to the inclusion and exclusion criteria. In addition, three neonates were excluded from further analysis because they did not receive the minimum established supplement dose (70% of the total prescribed dose, Figure 1). Successful supplementation was achieved in 18 infants, who constitute the study population in the current subsequent analyses.

After obtaining informed consent, infants were allocated by the neonatologist using a randomization list by emulsion groups: (1) 80:40 mg/kg/day ARA:DHA (80:40 group; *n* = 9) and (2) 120:60 mg/kg/day ARA:DHA (120:60 group; *n* = 9). Multiple birth infants were subjected to individual randomization.

### Experimental supplement product

The experimental supplement product was an emulsified oil containing ARA and DHA as triacylglycerol. The oil formulation was Formulaid^®^ (DSM Nutritional Products, Basel, Switzerland). This formula was treated under optimized conditions with an emulsifier (Aquacelle^®^, Pharmako Biotechnologies Pty Ltd, Frenchs Forest, Australia).

Emulsification of Formulaid^®^ with Aquacelle^®^ 4:1 and subsequent sonication 1:1 with human milk for enteral administration was done under the supervision and guidance of Prof. Carlos Torres, an expert in lipid emulsions (Institute of Food Science Research Food research center, CIAL, Madrid, Spain). Sonication was performed in cold water bath under an atmosphere of nitrogen and protected from light for 9 min with stop intervals to keep the sample cool. This procedure allowed to reduce the particle size (90% of the particles were < 1.89 μm) to produce fine and stable emulsions that improve the bioavailability of ARA and DHA in nutritional formulas.

To prevent oxidation of the emulsified oil, the experimental medicinal product was packaged in opaque bottles with special caps under nitrogen. The emulsification was performed every 7 days and stored at 4°C until use. When bottles were opened, they were discarded after 7 days.

### Supplementation protocol and nutritional intake variables

Supplementation with ARA:DHA emulsified oil was started as soon as the infant tolerated 20 ml/kg/day of enteral feeding and continued until 36 WPA or discharge, whichever occurred earlier. Supplements were given in three daily doses (9, 18, and 24 h) and were administered by the nurse through the orogastric tube or by mouth immediately prior to enteral feed. Thus, nutrition was used as a bolus to deliver the supplement. The supplementation was adjusted according to the infant’s body weight.

Nutritional intake measures included the day of life for the enteral nutrition initiation and the day at which enteral feedings increased with human milk (breast milk and/or donor milk). Infants were given parenteral nutrition within the first day of life until exclusive enteral nutrition (>120 ml/kg/day) was achieved. Prescription of parenteral and enteral nutrition was at the attending neonatologist’s decision. All enrolled infants received lipids with SMOFlipid (Fresenius Kabi AB, Uppsala, Sweden). Days of life when exclusively enteral nutrition and lipid supplementation initiation were taken were also collected. Additionally, the enteral nutritional status of premature neonates related to maternal own milk, donor breast milk and formula at days 7, 28, and 36 WPA in ml/kg/day was calculated.

### Blood extraction and plasma, whole blood, and buccal swabs collection

Blood was collected in EDTA tubes (Vacutainer^®^, Madrid, Spain) by venipuncture at birth (before supplementation, baseline), at 21 days of life and 36 WPA or discharge (end of supplementation). Blood samples were centrifuged (2,100 × *g*, 10 min at 4°C) to obtain plasma and stored at −80°C until use.

At the same time, fresh whole blood was spotted on PUFA-coated paper and air-dried in the dark for 30 min and stored at –20°C until oxylipin analysis.

The scraping samples of the oral mucosa were obtained at 36 WPA by the nursery staff. Infants did not consume any milk 1 h prior to providing the buccal swab specimen. A cotton swab was applied gently to both cheeks up and down 10 times with a double-tipped, sterile BBL™ CultureSwab™ EZ swab (BD Company, Franklin Lakes, NJ, United States). The swab was removed and placed back in the sterile tube. The tubes were preloaded with stabilizing solutions and immediately sent to the laboratory for analysis.

### Social and clinical data

Sociodemographic variables were collected through a standardized maternal interview, including maternal age (years), ethnicity (Caucasian/others), and educational level (illiterate/middle school/high school/university). Clinical variables were recorded from the medical records: assisted reproduction techniques (yes/no), multiple pregnancy (yes/no), DHA supplementation during pregnancy (yes/no), type of delivery (vaginal/C-section), chorioamnionitis (defined as maternal fever > 38°C in the 24 h prior to delivery, uterine tenderness, or leukocytosis of >15,000/mm^3^), and gestational age (completed weeks^+*days*^). The neonatal variables collected were sex, Apgar scores at 1 and 5 min, Score for Neonatal Acute Physiology II at birth, neonatal need intubation at birth, and arterial pH.

Short-term morbidity included BPD, late-onset sepsis (LOS), NEC, patent ductus arteriosus (PDA), and ROP. BPD was defined as a requirement for supplemental oxygen for ≥28 days categorized as low/moderate/severe lung disease if required oxygen therapy at 36 WPA. LOS was defined as clinical findings; increased number and severity of apneas, oxygen therapy without infectious lung; hypotension and/or tachycardia, increased time of the capillary filling, increased C-reactive protein (>30 mg/L), leukocytosis (>15,000/mm^3^), leukopenia (<3,000/mm^3^), and/or immature neutrophils/total neutrophils >0.12. NEC diagnosis based on clinical signs and radiological findings – Bell’s stages 2 and 3 – were considered definitive disease. PDA was defined based on the need to treatment, surgically or pharmacologically, by clinical and functional echocardiographic criteria. ROP was categorized according to the International Classification of Retinopathy of Prematurity (24).

Use of steroid (yes/no), surfactant (yes/no), days in caffeine treatment, days of diuretics, days on continuous positive airway pressure (CPAP), NICU stay duration (days), gestational age at discharge (completed weeks^+*days*^), and mortality were also collected.

The cerebral ultrasound scans (cUS) were performed at 36 WPA (Canon^®^, Aplio 500; transducer set at 8–10 MHz). All linear measurements on cUS were blinded and performed by one of the research teams (MY). The cUS measurements were obtained on the following planes: mid-coronal plane at the level of the foramina of Monro (anterior horn width, ventricular index, ventricular–cerebral ratio, frontal white matter height, interhemispheric fissure and subarachnoideal space, coronal atrial plane [mid-body ventricular width]), mid-sagittal plane (*corpus callosum* width and length, corpus *callosum-fastigium* length, *vermis* height, and A-P width), parasagittal plane through lateral ventricles (thalamus-occipital distance of lateral ventricles), and mastoid view (coronal transverse cerebellar diameter). The following cUS variables were collected: intraventricular hemorrhage, post-hemorrhagic ventriculomegaly, cerebellar hemorrhage, and white matter injury (25).

Brain function was assessed at 36 WPA using amplitude-integrated electroencephalography (aEEG). One-channel (P3–P4) aEEG traces were recorded for at least 4 h with the NicoletOne™ EEG System monitor (Natus Medical Inc., CA, United States). Tracings were evaluated following the brain maturation scoring system developed by Burdjalov et al. (26).

Noninvasive pulse oximetry SatO_2_/FiO_2_ ratio, as a ratio between peripheral oxygen saturation by pulse oximetry (Masimo Tech., Irvine, CA, United States) and fraction of inspired oxygen (FiO_2_, by ventilator, CPAP devices, or oximeters), was registered as well.

### Neonatal anthropometry and growth data

Weight (g), length (cm), and head circumference (HC, cm) were recorded at birth, 21 days, and 36 WPA or discharge. The Z-scores were calculated through reviewed Fenton growth curves (27). Weight, length, and HC gain were calculated as 36 WPA – at birth. In addition, growth velocity was calculated by the following equation. Weight velocity is reported as g/kg/day and length and HC as cm/day.


(1) Weightvelocity=1000L*n(Weightat 36WPAWeightatbirth)Numberofdays

(2) LengthandHCvelocity=(LengthorHCat 36WPALengthorHCatbirth)Numberofdays

### Long-chain polyunsaturated fatty acid analysis in plasma

A total of 200 μl of plasma samples were prepared directly to determinate the fatty acid methyl esters (FAME), without prior lipid extraction, following the acid–base methylation method described by Castro-Gomez et al. (28). Then, 75 μl of tritridecanoin (1.0 mg/ml, Sigma, St. Louis, MO, United States) was added to the samples as internal standard. Two independent methylation processes were carried out for each sample.

Long-chain polyunsaturated fatty acid analysis was carried out using an Autosystem chromatograph (Perkin Elmer, Beaconsfield, United Kingdom) fitted with a flame ionization detector. Helium was the carrier gas with a column inlet pressure set at 20 psig and a split ratio of 1:20. The injection volume was 0.5 μl of sample. The column (VF-23 ms, fused-silica capillary column, 30 m × 0.25 mm i.d. × 0.25 μm film thickness, Varian, Middelburg, Netherlands) was held at 60°C for 1 min after injection. Then, temperature-programmed at 10°C/min to 130°C, then at 3°C/min to 170°C, and finally at 10°C/min to 230°C, where it was held for 5 min. The injector and detector temperatures were set at 250 and 270°C, respectively. For stocktickerFAME identification and quantification, a stocktickerFAME 36 standard mixture (Larodan, Barcelona, Spain) was employed. The LCPUFAs are expressed as %nmol.

The change in LCPUFA levels at 36 WPA was calculated for each individual as the increment (Δ) of the subtraction between the final level at 36 WPA and the first level at birth.

### Oxylipin and free fatty acid analysis in whole blood

All free fatty acid and oxylipin analyses were undertaken in the fatty acid laboratory at the South Australian Health and Medical Research Institute. Free fatty acids and oxylipins were screened in dried blood spots using ultra-high-performance liquid chromatography (Agilent 1290 Infinity, Agilent Technologies, CA, United States) equipped with tandem mass spectrometry (Triple Quad 5500 system, AB SCIEX, MA, United States). Briefly, a 6-mm disk of blood was obtained from the dried blood PUFA-coat card and placed into a 96-well plate with extraction solvent (150 μl of 80% aqueous methanol) containing internal standards (0.1 ng/μl of d5-DHA and d8-AA; 0.05 ng/μl d5-EPA; 0.7 ng/μl of d5-ALA; 1 ng/μl of d4-LA; 0.01 ng/μl of d8–12S-hydroxy-eicosatetraenoic acid [HETE], d4-leukotriene B4 [LTB4], and d4–13S- hydroxy-octadecadienoic acid [HODE]). The plate was covered and gently shaken for 30 min at room temperature. The extraction from each well was transferred to a fresh well in a new plate, sealed, and placed in the ultra-high-performance liquid chromatography-tandem mass spectrometry system. Analytes were captured using multiple reaction monitoring conditions of the target parent and product ion as previously described (29). Seven free fatty acids and 21 oxylipins were analyzed. The free fatty acids are expressed in μg/ml and oxylipins in ng/ml.

The change in oxylipin levels at 36 WPA was calculated by subtracting, for each infant, the levels at birth from the levels at the end of supplementation.

### Genomic DNA extraction and single-nucleotide polymorphism detection

The neonatal buccal swab was used to detect the SNPs of the *FADS1* gene. The scraping samples were processed from the swab from each cheek using the commercial NúcleoSpin Tissue Kit (Macherey-Nagel, Cultek, Madrid, Spain), obtaining a final DNA yield > 20 ng/μl. Two SNPs located in the *FADS1* region were selected based on their association with LCPUFA levels in previous studies (30, 31). The oligonucleotides were designed for the SNPs with the last nucleotide unpaired for each allelic variant of the SNP (Supplementary Table 1). The PCR was performed using Promega 2X Master Mix (Promega Biotech Ibérica, SL, Madrid, Spain) following the instructions of the manufacturer. Subsequently, the electrophoresis in agarose gel was performed to visualize the results. All samples were verified by Sanger sequencing (Thermo Fisher Scientific, Madrid, Spain).

### Statistical analysis

It was considered to use nonparametric methods due to the small sample size. Therefore, quantitative variables are expressed as median and interquartile range [Q1; Q3], and qualitative variables are expressed as a relative frequency (%) and sample size (n). Mann–Whitney U-test was used to analyze the differences in the variables according to diet supplementation. Fisher’s exact test was used to explore the association between qualitative variables. The *p*-values were adjusted for false discovery rate using Benjamini–Hochberg approach, and statistical significance was considered at *p* < 0.05.

Statistical analysis was performed using the R software [version 3.5.2, R Core Team, (32)] within RStudio (version 1.1.453, RStudio, Inc., Vienna, Austria) using the *tidyverse, ggplot2, ggpubr, rio, CompareGroup*, and *multcomp* packages.

## Results

### Baseline characteristics

The maternal characteristics, including DHA supplement during pregnancy, were similar between groups. In addition, in neonatal characteristics at birth no significant differences between groups were found. The neonatal nutritional intake variables were not significant between groups (Supplementary Table 2). The timing for initiation of enteral nutrition, at achieved exclusive enteral nutrition and intravenous lipid supplementation, was similar among groups (Table 1). There was no evidence of intolerance to the supplement or palatability issues.

**TABLE 1:** Maternal and neonatal characteristics, and nutritional intakes according to the ARA:DHA group supplementation.

Maternal characteristics	80:40 group	120:60 group	*P*
Maternal age (years)	35.0 [33.5; 37.8]	35.0 [32.0; 37.0]	0.78^a^
Ethnicity			
Caucasian	8 (88.8%)	9 (100.0%)	0.99^b^
Others	1 (11.1%)	0 (0.0%)	
Education level			
Illiterate	0 (0.00%)	2 (22.2%)	0.61^b^
Middle school	1 (12.5%)	1 (11.1%)	
High school	3 (37.5%)	1 (11.1%)	
University	4 (50.0%)	5 (55.6%)	
Assisted reproduction techniques	3 (30.0%)	3 (27.3%)	0.99^b^
Multiple gestation	3 (30.0%)	4 (36.4%)	0.99^b^
DHA supplement during pregnancy	2 (25.0%)	5 (62.5%)	0.32^b^
C-section	7 (70.0%)	7 (63.6%)	0.99^b^
Chorioamnionitis	6 (60.0%)	4 (36.4%)	0.40^b^

**Neonatal characteristics**	**80:40 group**	**120:60 group**	***P***

Male sex	6 (60.0%)	4 (36.4%)	0.40^b^
Gestational age (completed weeks^+*days*^)	28^+6^ [27^+3^; 30^+3^]	29^+6^ [27^+3^; 30^+5^]	0.83^a^
Apgar score at 1 min	6.0 [3.3; 8.0]	6.0 [6.0; 8.0]	0.67^a^
Apgar score at 5 min	7.5 [6.25; 8.8]	8.0 [7.5; 9.0]	0.42^a^
Score for Neonatal Acute Physiology II	24.0 [4.75; 35.0]	0.0 [0.0; 15.5]	0.11^a^
Intubation at birth	2 (20.0%)	1 (9.1%)	0.59^b^
Arterial pH	7.3 [7.2; 7.3]	7.3 [7.3; 7.4]	0.08^a^
Enteral nutrition initiation (days of life)	1.5 [1.0; 2.0]	1.0 [1.0; 2.0]	0.48^a^
Overcoming of trophic nutrition (days of life)	3.0 [2.0; 3.0]	3.0 [2.0; 3.0]	0.79^a^
Exclusively enteral nutrition (days of life)	10.5 [7.5; 12.0]	7.0 [6.0; 9.0]	0.18^a^
Lipid supplementation initiation (days of life)	3.0 [2.3; 3.0]	3.0 [3.0; 4.0]	0.30^a^
Gestational age at discharge (completed weeks^+*days*^)	36^+6^ [36^+0^; 38^+2^]	37^+0^ [36^+2^; 37^+1^]	0.85^a^
Neonatal intensive care unit duration (days)	18.0 [6.0; 33.0]	14.0 [12.5; 23.0]	0.97^a^

Data show median and interquartile range [Q1; Q3] in quantitative variables and sample size (*n*) and relative frequency (%) in qualitative variables. The *p*-value was extracted from ^a^Mann–Whitney *U*-test or ^b^Fisher’s exact test.

### Plasma and free long-chain polyunsaturated fatty acid levels with arachidonic acid:Docosahexaenoic acid supplementation

The baseline plasma LCPUFA levels did not show statistical differences between groups. Additionally, no statistically significant differences were detected at 21 days and 36 WPA in DHA and ARA plasma levels between groups (Supplementary Table 3).

Nevertheless, the change from baseline to 36 WPA for ARA levels was in the 120:60 group = 1.68 [1.38; 3.16] %nmol and in the 80:40 group = 0.15 [−0.67; 0.69] %nmol. This increment was statistically significant, while the increment was not significant in DHA levels (Table 2). In whole blood, the change from baseline to 36 WPA for free ARA levels was in the 120:60 group = –1.21 [–1.96; 2.58] μg/ml and in the 80:40 group = –4.22 [−5.97; −4.13] μg/ml. Being consistent with plasma, the change in whole blood was statistically significant (Table 2).

**TABLE 2:** Changes in total plasma and free whole blood LCPUFAs from birth to 36 weeks’ postmenstrual age according to the ARA:DHA group supplementation.

Fatty acids in plasma (%nmol)	80:40 group	120:60 group	*P*
Linoleic acid (LA)	8.87 [8.07; 13.3]	8.10 [6.89; 11.2]	0.76
Dihomo-γ-linolenic acid (DGLA)	−0.10 [−0.26; 0.44]	−0.05 [−0.22; 0.31]	0.69
Arachidonic acid (ARA)	0.15 [−0.67; 0.69]	1.68 [1.38; 3.16]	**0.031**
α-Linolenic acid (ALA)	0.22 [0.00; 0.33]	0.00 [0.00; 0.26]	0.58
Eicosapentaenoic acid (EPA)	−0.17 [−0.40; 0.07]	0.00 [−0.20; 0.33]	0.27
Docosahexaenoic acid (DHA)	−0.12 [−0.35; 0.16]	−0.14 [−0.37; 0.33]	0.90
ARA:DHA	0.25 [−1.96; 1.18]	0.43 [0.21; 2.85]	0.13

**Free fatty acids in whole blood (μg/ml)**	**80:40 group**	**120:60 group**	***P***

Linoleic acid (LA)	0.10 [−3.85; 0.57]	2.08 [0.97; 5.16]	0.18
Dihomo-γ-linolenic acid (DGLA)	−0.10 [−0.30; 0.49]	−0.10 [−0.30; 0.29]	0.63
Arachidonic acid (ARA)	−4.22 [−5.97; −4.13]	−1.21 [−1.96; 2.58]	**0.025**
α-Linolenic acid (ALA)	−0.59 [−1.41; 0.44]	0.52 [0.30; 1.16]	0.30
Eicosapentaenoic acid (EPA)	−0.40 [−0.58; −0.29]	−0.04 [−0.05; −0.02]	0.05
Docosahexaenoic acid (DHA)	−2.30 [−3.13; −1.97]	0.06 [−0.46; 0.51]	0.10
ARA:DHA	−0.25 [−0.82; 0.41]	0.09 [−1.27; 1.12]	0.66

Data show median and interquartile range [Q1; Q3]. The *p*-value was extracted from Mann–Whitney U-test. Bold indicates *p* < 0.05.

### Oxylipin levels with arachidonic acid:Docosahexaenoic acid supplementation

**FIGURE 2:**
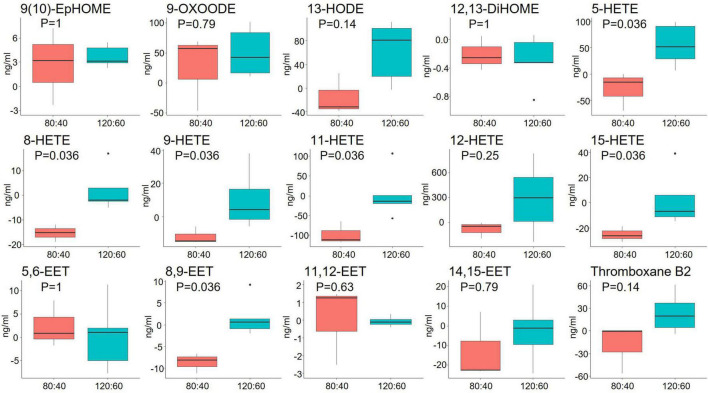
Changes in whole blood oxylipins derived from omega-6 LCPUFAs from birth to 36 weeks’ postmenstrual age according to ARA:DHA group supplementation. Data show median and interquartile range [Q1; Q3]. The *p*-value was extracted from Mann–Whitney U-test.

The n-6 LCPUFA-derived oxylipin levels, except for the 5-HETE level that was lower at birth in the 120:60 group than the 80:40 group, did not show statistical differences between groups neither at birth nor at 21 days and 36 WPA (Supplementary Table 4). However, considering the change from baseline to 36 WPA, the 120:60 group showed significantly higher ARA-derived oxylipins: 5-, 8-, 9-, 11-, 15-HETE and 8,9-EET levels than the 80:40 group (Figure 2).

**FIGURE 3:**
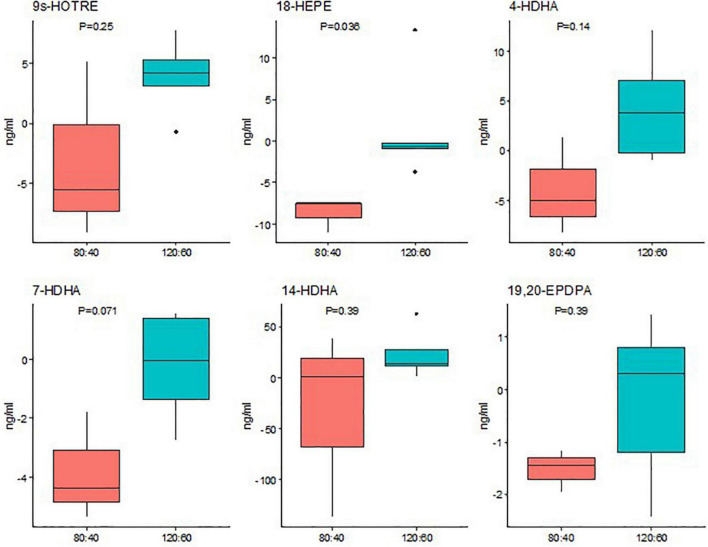
Changes in whole blood oxylipins derived from omega-3 LCPUFAs from birth to 36 weeks’ postmenstrual age according to ARA:DHA group supplementation. Data show median and interquartile range [Q1; Q3]. The *p*-value was extracted from Mann–Whitney U-test.

The n-3 LCPUFA-derived oxylipin concentrations, except for the 4-HDHA level that was lower in the 120:60 group at birth and higher at 36 WPA than the 80:40 group, did not show statistical differences between groups neither at birth nor at 21 days and 36 WPA (Supplementary Table 5). However, considering the change from baseline to 36 WPA, the 120:60 group showed significantly higher EPA-derived oxylipin, 18-HEPE concentrations than the 80:40 group (Figure 3).

### Interaction between single-nucleotide polymorphisms with arachidonic acid:Docosahexaenoic acid supplementation

The effects between alleles of SNPs and supplementation group were examined. In the rs174545 SNP, the genotype CC was randomly represented only in the infants in the 120:60 group (*n* = 3), and the genotype GC was only represented in the infants in the 80:40 group (*n* = 1). Furthermore, in the rs174546 SNP, the genotype CC was represented in three infants in the 80:40 group and one in the 120:60 group. Additionally, the genotype TT was represented in one infant in the 80:40 group.

Considering the low proportions, the CC genotype of rs174545 SNP and CT genotype of rs174546 showed higher DHA levels when received 60 mg/kg/d DHA compared to CG genotype of rs174545 SNP and TT genotype of rs174546, respectively (Supplementary Figure 1).

### Neonatal anthropometry and clinical variables with arachidonic acid:Docosahexaenoic acid supplementation

**FIGURE 4:**
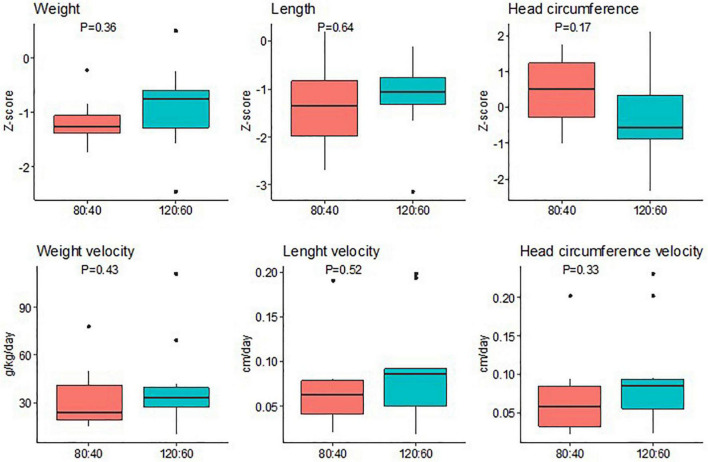
Neonatal differences in Z-score between 36 weeks’ postmenstrual age and birth and growth velocities in weight, length, and head circumference according to the ARA:DHA group supplementation. Data show median and interquartile range [Q1; Q3]. The *p*-value was extracted from Mann–Whitney U-test.

The neonatal anthropometry parameters did not show statistical difference at any time between groups (Supplementary Table 6). In addition, weight, length, and JC gain in Z-scores and growth velocities did not show statistically significant differences between groups (Figure 4).

There were no differences in neonatal clinical outcomes between groups, premature comorbidities (BPD, LOS, NEC, PDA, and ROP), and noninvasive pulse oximetry SatO_2_/FiO_2_ ratio (Table 3).

**TABLE 3:** Neonatal clinical outcomes according to the ARA:DHA group supplementation.

	80:40 group	120:60 group	*P*
Use of steroids	3 (30.0%)	1 (9.1%)	0.31^a^
Use of surfactant	2 (20.0%)	1 (9.1%)	0.59^a^
Bronchopulmonary dysplasia	4 (40.0%)	3 (27.3%)	0.66^a^
Level of bronchopulmonary dysplasia			
Low	1 (25.0%)	1 (33.3%)	0.99^a^
Moderate	2 (50.0%)	2 (66.7%)	
Severe	1 (25.0%)	0 (0.0%)	
Use of caffeine (days)	34.5 [20.2; 53.2]	34.0 [22.0; 47.5]	0.83^b^
Use of diuretics (days)	0.5 [0.0; 13.2]	0.0 [0.0; 0.5]	0.23^b^
CPAP (days)	10.5 [2.0; 16.0]	4.00 [2.5; 5.0]	0.38^b^
SatO_2_/FiO_2_	452 [335; 471]	466 [457; 471]	0.60^b^
Late onset sepsis	4 (40.0%)	2 (18.2%)	0.36^a^
Necrotizing enterocolitis	1 (10.0%)	2 (18.2%)	0.99^a^
Patent ductus arteriosus	0 (0.0%)	2 (18.2%)	0.48^a^
Retinopathy of prematurity	1 (10.0%)	2 (18.2%)	0.99^a^
Intraventricular hemorrhage	1 (10.0%)	0 (0.0%)	0.99^a^
Post-hemorrhagic ventriculomegaly	1 (10.0%)	0 (0.0%)	0.99^a^
Cerebellar hemorrhage	0 (0.0%)	0 (0.0%)	–
White matter injury	1 (10.0%)	1 (9.1%)	0.99^a^
Subarachnoid space (mm)	2.0 [1.7; 2.7]	1.2 [0.0; 1.4]	**0.041^b^**

Data show median and interquartile range [Q1; Q3] in quantitative variables and sample size (*n*) and relative frequency (%) in qualitative variables. The *p*-value was extracted from ^a^Fisher’s exact test or ^b^Mann–Whitney U-test. Bold shows *p* < 0.05. Continuous positive airway pressure (CPAP); oxygen saturation (SatO_2_); fraction of inspired oxygen (FiO_2_).

Regarding neurological outcome variables, all patients had the same Burdjalov score at 36 WPA (13 [13; 13]). No differences were found between groups in ultrasonographic variables of brain damage. All the linear measurements of brain growth were similar between both groups; however, subarachnoid space was significantly greater in the infants in the 80:40 group compared to the infants in the 120:60 group (Table 3).

## Discussion

Optimal nutrition is critical to support adequate development in very-low-birth-weight infants. Our data demonstrate that the supplementation at higher doses (120:60 ARA:DHA) raises plasma levels of ARA and its derived oxylipins, hydroxyeicosatetraenoic acids (HETEs) and epoxyeicosatrienoic acid (EET) at 36 WPA. At the same time, this intervention also elevated EPA-derived oxylipins, 18-hydroxyeicosapentaenoic acid (18-HEPE). The results support that ARA levels, the most predominant LCPUFA in human milk, are intake dependent.

Previous studies have focused on increasing DHA content in mother’s milk or infant formula, achieving a higher infant DHA level in plasma and erythrocytes. Smithers et al. (DINO trial) found that preterm infants fed with milk containing 1% of DHA vs. 0.3% of DHA increased their plasma and erythrocyte DHA levels by ∼30% (33). This finding was consistent with other trials in preterm infants (34, 35). However, extremely premature infants typically do not reach full enteral feedings until several weeks and standard available parenteral lipid emulsions do not provide this preformed LCPUFA; therefore, blood levels decline rapidly after birth (16). Consequently, many supplementation strategies are centered on increasing DHA plasma levels using a concentrated DHA supplement. Baack et al. proposed a new DHA supplementation strategy. Those preterm infants who received enteral DHA supplements (50 mg/day) had a progressive increased in DHA plasma levels, but lower than term peers (36). Afterward, Collins et al. administered DHA as an emulsion (at doses 40, 80, and 120 mg/kg), finding that higher DHA supplementation was directly related to blood DHA levels (23). Previous studies using nonemulsified fatty acids have not been able to show differences in fatty acid levels (37). Although our emulsion has a small particle size (90% of the particles were smaller than 1.89 μm), *in vitro* digestion tests have shown limited digestibility that limits bioavailability, which may explain why higher differences were not found between the two groups. In studies that only supplement DHA, ARA, and LA levels dropped off proportionally (23). This may explain why no clear long-term benefits or harm were demonstrated for preterm infants receiving LCPUFA-supplemented formula (38). To avoid these blood-level declines, other studies have added ARA to supplementation, obtaining higher LCPUFA blood levels and better results in growth and psychomotor development in very preterm infants (39, 40). The balance between n-6 and n-3, and their metabolites, is important and highly complex, particularly in preterm infants (9). Our results also support the importance of ARA supplementation.

Human milk contains the essential fatty acids LA and ALA and their respective n-6 and n-3 LCPUFA metabolites, ARA and DHA. Additionally, it has been demonstrated that human milk ARA levels are higher with increased ARA supplementation (41). Although several studies in very preterm infants have shown a decrease in LCPUFA levels compared to term infants (3, 42) and existing evidence supports LCPUFAs supplementation in these infants (43), doses, the route of administration, and the choice of better LCPUFA supplementation remain unknown. According to our data, 120:60 ARA:DHA supplementation increased DHA metabolite levels compared to lower doses (80:40 ARA:DHA), without a significant increase in DHA plasma levels. ARA and DHA are important components of cell membranes, possibly our intervention increases LCPUFA levels in cell membranes, but it is not reflected in plasma. However, this hypothesis needs further corroboration.

The data show that dietary DHA and ARA can dose-dependently modulate endogenous n-3 and n-6 oxylipin levels. Increasing ARA intake raised plasma ARA levels accompanied with whole blood 8-, 9-, 11-, 15-HETE and 8,9-EET concentrations. The dose-dependent increases in these oxylipins suggest increased conversion of ARA *via* the cytochrome P450/soluble epoxide hydrolases (CYP450/sEH) enzymatic pathway. Despite not finding significant differences in DHA plasma levels, dietary DHA also increased the EPA-derived oxylipin 18-HEPE metabolized by CYP450/sHE (44), suggesting that DHA retroconversion to EPA may occur to some extent, as other authors reported (45). Due to competitive metabolic effects between n-3 and n-6 LCPUFAs (46), the effect of retroconversion of DHA to EPA, and their subsequent metabolites, may be less pronounced when ARA is concomitantly supplemented. This could demonstrate the oxylipin levels may not necessarily correlate LCPUFA levels. Therefore, it is important to analyze the oxylipin plasma levels because they are the main mediators of LCPUFA effects in the human physiology. 18-HEPE is a precursor of E-series resolvin (RvE) pathway. Thus, both RvE2 and 18-HEPE can act as pro-resolving mediators of inflammation showing reduction of neutrophil chemoattraction and enhancement macrophage phagocytosis (47). In addition, EPA-derived oxylipins, particularly HEPEs and resolvins, provide a chance to fight against chronic inflammatory diseases and sepsis (48).

At least, part of the beneficial health effects of ARA and DHA is mediated by their oxylipins or shifts in the overall oxylipin profile. However, it is still unknown which of these oxylipins may mediate beneficial health effects. The EETs are synthesized from ARA by epoxygenase enzymes being produced by neural and vascular tissues, among others. Data demonstrate manifold biological activities of EETs, including anti-inflammatory, neuroprotective effects, vasodilation, endothelial proliferation, migration, and angiogenesis. In particular, 8,9-EET has a prominent role in vascular regulation (49). As we found no differences in neonatal comorbidities, more research is needed to relate the effects on the health of preterm infants. However, our supplementation with high doses of ARA:DHA points to an increase in the blood of 18-HEPE and 8,9-EET oxylipins.

Recent studies had supplemented preterm infants with a high dose of LCPUFAs (120 mg/kg/day of DHA and 240 mg/kg/day of ARA) and found higher LCPUFA blood levels than those with lower doses (20). Benefits of LCPUFA supplementation in extremely premature infants appear promising, but not yet conclusive, partly due to variability in supplementation methods. We found no statistical differences in neonatal clinical outcomes between groups. Although our study was not designed to analyze these variables and the sample was small to draw conclusions, we did not observe an increased risk of BPD, as has been previously described in other cohorts (50) or in other comorbidities (9, 51). In extremely preterm infants, low DHA and ARA blood levels have been associated with increased early systematic inflammation (52). This inflammation plays a role in the development of intraventricular hemorrhage and white matter injury. In this study, even though brain imaging did not show a difference in damage variables between groups, a decrease in the subarachnoid space was observed in those in the 120:60 group. This could suggest greater brain growth in preterm infants supplemented with a higher dose of ARA:DHA. This affirmation could be supported due to other trials having associated with a higher LCPUFA blood levels with a decrease in intraventricular hemorrhage (53) and greater brain volume (54). In addition, an increase in LCPUFA and oxylipin levels to promote growth is also possible, mainly in the length of preterm infants. The growth could modulate long-term neonatal morbidities outcome. Future studies with a larger sample size and improved absorption and bioavailability of lipid supplementation are needed to affirm these conclusions.

In contrast, the SNP data from *FADS1* gene could suggest that minor allele carriers had lower DHA and that supplementation may only significantly increase DHA in major allele carriers. The specific findings according to the *FADS1* gene show biological plausibility, with *FADS1* encoding for the Δ5-desaturase enzyme. Specifically, desaturates dihomo-γ-linoleoate and eicosatetraenoate to generate ARA and EPA, respectively (55, 56). This data could show that in our cohort the endogenous synthesis plays an important role.

Currently, benefits of ARA:DHA supplementation are promising but unclear. Studies using lipid supplementation appear to have higher and closer LCPUFA blood levels to term infant, which could lead to improved clinical outcomes and neonatal prognosis. Nevertheless, our study is not without its limitations. The sample size was particularly small, and we cannot rule out the possibility to detect any true effects. A control group was not included, although no differences were found in a previous publication between the 80:40 group and controls (19). Therefore, our study requires replication by larger randomized clinical trials to support the safe and effective dose of an emulsified ARA:DHA supplementation in preterm infants and confirm the findings.

## Conclusion

In very preterm infants, the ARA:DHA supplementation with the triacylglycerol emulsion at a high dose (120:60 mg/kg/day) increased ARA levels, ARA- and EPA-oxylipins derived, particularly in whole blood, from birth to 36 WPA compared to low-dose supplementation (80:40 mg/kg/day), without recursion in the intolerance or palatability. Additionally, no differences in comorbidities rate were detected.

## Data availability statement

The original contributions presented in this study are included in the article/Supplementary material, further inquiries can be directed to the corresponding author.

## Ethics statement

The studies involving human participants were reviewed and approved by the Hospital Universitario La Paz Ethics Committee (Ref. HULP-4840). Written informed consent to participate in this study was provided by the participants or their legal guardian/next of kin.

## Author contributions

PÁ contributed to the design, recruitment of infants, acquisition and interpretation of data, and drafted the manuscript. DR-C performed the statistical analysis and interpretation of the data, and drafted the manuscript, tables, and figures. MTM contributed to the elaboration of the emulsion and collection of samples. BM contributed to the acquisition of data. MVC and GL performed the biochemical analysis of the samples. AER, MY, and MC contributed to the acquisition and interpretation of data. MCM and AS-P contributed to the design and performed the analysis of the samples. EV contributed to the interpretation of the data. JF and RG contributed to the biochemical analysis and drafted the manuscript. MSdP contributed to the conception, design, recruitment, acquisition, interpretation, drafting, and review of the manuscript. All authors contributed to the article and approved the submitted version.
