# Supplementary material for: Randomized controlled trial of early arachidonic acid and docosahexaenoic acid enteral supplementation in very preterm infants
Source: Front Pediatr. 2022 Aug 25;10:947221. doi: 10.3389/fped.2022.947221 (PMC9452757; doi:10.3389/fped.2022.947221)

Supplementary Material

Table S1. Oligonucleotides for single nucleotide polymorphisms (SNPs) detection of *FADS1* gene.

SNPs	Primer	Sequence
rs174545	Forward	CCCCAATACCACAGTCTTGA
	Reverse C	GCTTCTCCATCCTCATTTGC
	Reverse G	CCTTCTCCATCCTCATTTGC
rs174546	Forward C	TCAAGCTCCCCTCTGCC
	Forward T	TCAAGCTCCCCTCTGCT
	Reverse	GACATGTAGGGTGGGGAGTG

Table S2. Enteral nutritional status of premature neonates according to ARA:DHA group supplementation.

	**80:40 group**	**120:60 group**	**P**
Maternal own milk (ml/kg/day)			
At 7 days	54.2 [15.4; 66.5]	53.6 [32.6; 144.0]	0.27
At 28 days	130.0 [0.0; 153.0]	131.0 [111.0; 154.0]	0.45
At 36 WPA	130.0 [88.4; 145.0]	87.5 [20.4; 153.0]	0.83
Maternal donor milk (ml/kg/day)			
At 7 days	0.0 [0.0; 41.2]	0.0 [0.0; 37.1]	0.77
At 28 days	0.0 [0.0; 14.4]	0.0 [0.0; 43.7]	0.51
At 36 WPA	-	-	-
Formula (ml/kg/day)			
At 7 days	-	-	-
At 28 days	0.0 [0.0; 25.9]	0.0 [0.0; 0.0]	0.20
At 36 WPA	0.0 [0.0; 59.0]	0.0 [0.0; 0.0]	0.38

Data shown median and interquartile range [Q1; Q3]. The p-value (P) was extracted from U Mann–Whitney test. Weeks postmenstrual age (WPA).

Table S3. Plasma fatty acids levels according to ARA:DHA group supplementation.

**Fatty acids (%nmol)**	**80:40 group**	**120:60 group**	**P**
Linoleic acid (LA)			
At birth	9.75 [7.25; 14.0]	11.3 [9.57; 12.0]	0.97
At 21 days	17.9 [17.0; 18.5]	14.6 [10.7; 17.0]	0.07
At 36 WPA	19.7 [17.2; 24.1]	19.8 [18.3; 20.9]	0.97
Dihomo-γ-linolenic acid (DGLA)			
At birth	1.18 [1.13; 1.41]	1.29 [1.13; 1.87]	0.51
At 21 days	1.46 [1.36; 1.52]	1.49 [1.35; 1.74]	0.63
At 36 WPA	1.43 [1.12; 1.47]	1.17 [1.08; 1.72]	0.97
Arachidonic acid (ARA)			
At birth	6.43 [5.04; 7.24]	5.95 [4.28; 6.60]	0.45
At 21 days	6.48 [5.65; 6.68]	7.49 [5.78; 7.90]	0.20
At 36 WPA	6.07 [4.96; 7.39]	7.42 [5.66; 8.42]	0.20
α-Linolenic acid (ALA)			
At birth	0.00 [0.00; 0.00]	0.00 [0.00; 0.00]	0.81
At 21 days	0.00 [0.00; 0.29]	0.00 [0.00; 0.37]	0.73
At 36 WPA	0.31 [0.00; 0.45]	0.00 [0.00; 0.44]	0.41
Eicosapentaenoic acid (EPA)			
At birth	0.40 [0.25; 0.56]	0.26 [0.00; 0.57]	0.62
At 21 days	0.23 [0.00; 0.56]	0.76 [0.22; 0.82]	0.11
At 36 WPA	0.20 [0.00; 0.32]	0.33 [0.23; 0.50]	0.23
Docosahexaenoic acid (DHA)			
At birth	1.52 [1.07; 1.82]	1.37 [1.17; 1.93]	0.69
At 21 days	1.15 [0.90; 1.38]	1.43 [1.28; 1.61]	0.17
At 36 WPA	1.27 [1.21; 1.40]	1.42 [1.22; 1.58]	0.51
ARA:DHA			
At birth	4.22 [3.86; 4.79]	3.39 [3.00; 5.79]	0.23
At 21 days	5.36 [4.69; 6.23]	5.15 [3.05; 6.08]	0.51
At 36 WPA	4.60 [3.89; 5.76]	5.84 [5.20; 6.91]	0.20

Data shown median and interquartile range [Q1; Q3]. The p-value (P) was extracted from U Mann–Whitney test. Weeks postmenstrual age (WPA).

Table S4. Omega-6 LCPUFAs derived oxylipin levels according to ARA:DHA group supplementation.

**Oxylipins (ng/ml)**	**80:40 group**	**120:60 group**	**P**		**80:40 group**	**120:60 group**	**P**
9(10)-EpHOME				12-HETE			
At birth	4.42 [4.35; 5.06]	2.33 [1.81; 3.50]	0.09	At birth	159 [112; 180]	103 [33.9; 139]	0.17
At 21 days	5.61 [3.71; 6.48]	4.66 [2.50; 5.56]	0.52	At 21 days	390 [115; 861]	337 [154; 493]	0.90
At 36 WPA	4.33 [3.25; 6.93]	4.79 [4.67; 7.67]	0.52	At 36 WPA	160 [154; 329]	224 [49.8; 632]	0.75
9-OXOODE				15-HETE			
At birth	54.6 [19.3; 62.4]	22.6 [18.1; 38.3]	0.22	At birth	35.2 [25.1; 43.1]	18.3 [13.1; 22.2]	0.09
At 21 days	58.8 [28.1; 124]	37.3 [25.3; 50.3]	0.52	At 21 days	13.8 [8.43; 18.6]	19.3 [11.1; 33.1]	0.44
At 36 WPA	56.3 [23.3; 103]	64.7 [41.5; 103]	0.75	At 36 WPA	16.0 [12.9; 17.0]	13.3 [7.72; 25.8]	0.75
13-HODE				5,6-EET			
At birth	88.5 [41.5; 97.2]	45.8 [41.1; 69.9]	0.47	At birth	3.30 [2.37; 4.09]	3.11 [1.93; 7.40]	0.94
At 21 days	115 [61.1; 218]	64.1 [49.1; 99.3]	0.20	At 21 days	4.07 [2.51; 4.58]	3.54 [2.27; 6.47]	0.90
At 36 WPA	106 [60.2; 137]	110 [73.7; 137]	0.63	At 36 WPA	6.56 [4.00; 8.52]	3.70 [1.43; 6.10]	0.26
12,13-DiHOME				8,9-EET			
At birth	1.19 [1.16; 1.43]	1.13 [0.99; 1.25]	0.57	At birth	9.83 [4.76; 13.0]	3.18 [2.88; 5.48]	0.12
At 21 days	1.01 [0.70; 2.03]	0.85 [0.76; 1.00]	0.61	At 21 days	5.27 [2.79; 8.38]	4.67 [3.63; 6.29]	0.80
At 36 WPA	0.85 [0.79; 0.98]	0.71 [0.63; 0.84]	0.34	At 36 WPA	3.70 [2.48; 4.75]	5.15 [2.48; 7.68]	0.52
5-HETE				11,12-EET			
At birth	148 [146; 163]	93.6 [81.8; 113]	**0.012**	At birth	0.97 [0.64; 2.60]	0.97 [0.69; 1.09]	0.81
At 21 days	101 [70.2; 155]	114 [91.4; 147]	0.80	At 21 days	1.17 [0.96; 1.69]	2.63 [1.46; 3.69]	0.12
At 36 WPA	117 [64.4; 136]	161 [123; 192]	0.34	At 36 WPA	1.47 [0.82; 2.43]	0.98 [0.83; 1.37]	0.63
8-HETE				14,15-EET			
At birth	20.9 [12.2; 23.4]	9.72 [6.89; 11.9]	0.09	At birth	26.9 [25.4; 37.9]	14.5 [12.6; 21.0]	0.09
At 21 days	11.0 [5.02; 15.4]	9.82 [8.83; 15.2]	0.52	At 21 days	13.4 [11.8; 14.5]	23.7 [16.1; 27.1]	0.37
At 36 WPA	8.53 [5.42; 8.98]	10.0 [5.09; 15.0]	0.63	At 36 WPA	19.5 [12.0; 30.6]	12.7 [11.8; 21.1]	0.75
9-HETE				Thromboxane B2 (TXB2)			
At birth	23.9 [13.7; 26.6]	12.0 [9.21; 13.4]	0.09	At birth	3.36 [2.56; 41.0]	2.30 [1.37; 5.38]	0.29
At 21 days	21.6 [9.27; 37.2]	18.8 [14.1; 25.2]	0.90	At 21 days	9.99 [4.69; 24.9]	5.17 [3.50; 7.04]	0.37
At 36 WPA	16.5 [9.60; 21.7]	15.7 [8.96; 28.2]	0.99	At 36 WPA	5.52 [4.15; 12.5]	13.9 [5.42; 34.2]	0.34
11-HETE							
At birth	123 [83.4; 130]	46.0 [42.4; 83.3]	0.12				
At 21 days	44.5 [28.4; 75.0]	58.0 [41.1; 86.6]	0.37				
At 36 WPA	49.7 [26.1; 56.7]	49.9 [30.7; 76.8]	0.52				

Data shown median and interquartile range [Q1; Q3]. The p-value (P) was extracted from U Mann–Whitney test. Weeks postmenstrual age (WPA). In bold is shown $P<0.05$.

Table S5. Omega-3 LCPUFAs derived oxylipin levels according to ARA:DHA group supplementation.

**Oxylipins (ng/ml)**	**80:40 group**	**120:60 group**	**P**
9s-HOTRE			
At birth	6.53 [2.93; 9.80]	2.59 [1.27; 4.51]	0.09
At 21 days	4.66 [3.10; 6.90]	3.64 [2.88; 6.18]	0.70
At 36 WPA	3.81 [3.01; 7.41]	6.19 [2.82; 8.28]	0.75
18-HEPE			
At birth	7.60 [0.60; 8.21]	1.19 [0.90; 3.83]	0.57
At 21 days	1.39 [0.52; 2.49]	1.67 [0.78; 2.91]	0.80
At 36 WPA	0.39 [0.22; 0.64]	0.64 [0.48; 2.07]	0.42
4-HDHA			
At birth	14.2 [10.5; 16.7]	9.49 [9.09; 11.3]	**0.028**
At 21 days	10.0 [8.69; 11.9]	9.45 [8.94; 17.0]	0.90
At 36 WPA	8.31 [6.84; 8.58]	13.6 [10.9; 15.8]	**0.025**
7-HDHA			
At birth	4.03 [2.69; 5.78]	3.07 [1.31; 3.36]	0.22
At 21 days	1.93 [1.00; 2.28]	1.07 [0.45; 2.02]	0.44
At 36 WPA	1.72 [0.72; 1.86]	2.16 [1.80; 3.17]	0.15
14-HDHA			
At birth	13.2 [4.35; 63.1]	16.6 [8.07; 20.3]	0.81
At 21 days	8.25 [6.90; 14.5]	13.0 [8.30; 27.1]	0.25
At 36 WPA	8.44 [5.75; 15.0]	18.6 [15.5; 31.7]	0.08
19,20-EPDPA			
At birth	1.68 [1.55; 2.50]	1.20 [0.82; 1.68]	0.12
At 21 days	1.81 [0.91; 2.29]	1.66 [1.01; 1.84]	0.30
At 36 WPA	0.81 [0.42; 1.66]	1.68 [1.22; 2.43]	0.34

Data shown median and interquartile range [Q1; Q3]. The p-value (P) was extracted from U Mann–Whitney test. Weeks postmenstrual age (WPA). In bold is shown $P<0.05$.

Table S6. Neonatal anthropometric parameters according to ARA:DHA group supplementation.

Anthropometry	80:40 group	120:60 group	P
Weight (g)			
At birth	1063 [940; 1412]	1210 [1086; 1468]	0.48
At 21 days	1334 [1193; 1597]	1360 [1212; 1775]	0.78
At 36 WPA	2255 [1905; 2336]	2350 [2088; 2372]	0.56
Length (cm)			
At birth	36.2 [34.4; 40.1]	38.5 [36.5; 39.2]	0.42
At 21 days	40.0 [37.0; 42.8]	39.5 [38.0; 43.0]	0.73
At 36 WPA	43.8 [43.1; 44.0]	44.0 [42.5; 45.0]	0.45
Head circumference (cm)			
At birth	25.0 [22.6; 26.8]	27.0 [25.0; 27.8]	0.14
At 21 days	27.2 [26.1; 28.6]	28.0 [26.8; 29.2]	0.55
At 36 WPA	31.5 [30.8; 32.8]	32.4 [31.6; 32.9]	0.56

Data shown median and interquartile range [Q1; Q3]. The p-value (P) was extracted from U Mann–Whitney test. Weeks postmenstrual age (WPA).

Figure S1. Effect of rs174545 and rs174546 SNPs on plasma ARA and DHA levels, and ARA:DHA ratio at 36 week of postmenstrual age according to ARA:DHA group supplementation. Data shown median and interquartile range [Q1; Q3]. The p-value (P) was extracted from U Mann–Whitney test in those cases where a comparison was possible.

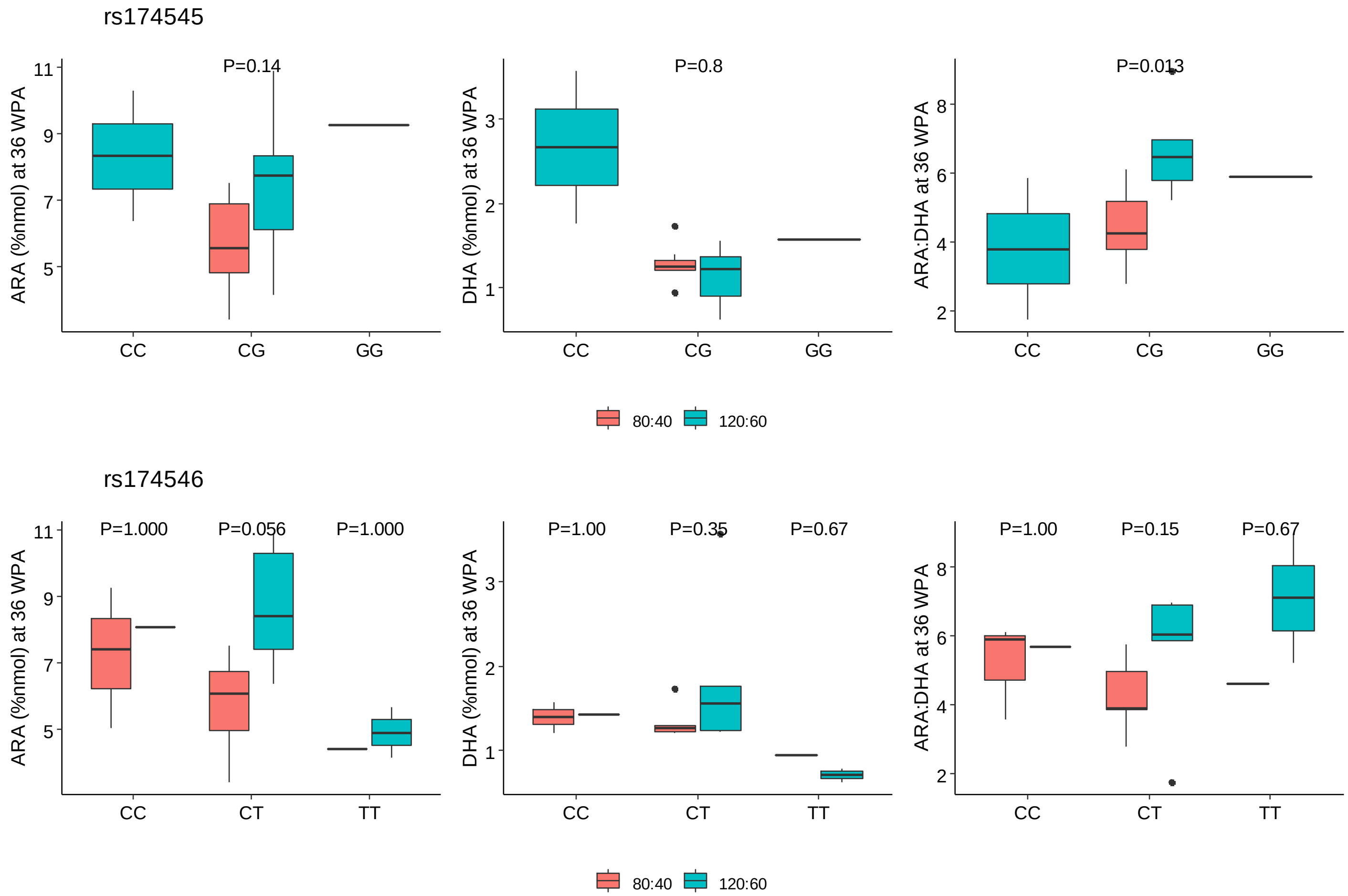

Supplement: Supplementary file 1 [file Data_Sheet_1.PDF]
